# Deciphering the Clinical Behaviour of Invasive Lobular Carcinoma of the Breast Defines an Aggressive Subtype

**DOI:** 10.3390/cancers16101893

**Published:** 2024-05-16

**Authors:** Shorouk Makhlouf, Nehal M. Atallah, Susanna Polotto, Andrew H. S. Lee, Andrew R. Green, Emad A. Rakha

**Affiliations:** 1 Nottingham Breast Cancer Research Centre, Academic Unit for Translational Medical Sciences, School of Medicine, University of Nottingham, Nottingham NG7 2RD, UK; shorouk.makhlouf@nottingham.ac.uk (S.M.); nehal.atallah@nottingham.ac.uk (N.M.A.); andrew.green@nottingham.ac.uk (A.R.G.); 2 Department of Pathology, Faculty of Medicine, Assiut University, Assiut 71515, Egypt; 3 Department of Pathology, Faculty of Medicine, Menoufia University, Menoufia 32928, Egypt; 4 Division of Oncoplastic Surgery, Nottingham Breast Institute, Nottingham University Hospitals, NHS Trust, Nottingham NG5 1PB, UK; susanna.polotto@nuh.nhs.uk; 5 Department of Histopathology, Nottingham University Hospitals, NHS Trust, Nottingham NG5 1PB, UK; 6 Department of Pathology, Hamad Medical Corporation, Doha 3050, Qatar

**Keywords:** breast cancer, invasive lobular carcinoma, variants, aggressive behaviour

## Abstract

**Simple Summary:**

Invasive lobular carcinoma represents a distinct histological type of breast cancer, characterised by morphological, genetic, and behavioural differences from other types. However, the behaviour of invasive lobular carcinomas is not uniform, and some tumours show poor outcome. This may attribute to the presence of aggressive variants. Therefore, we performed detailed investigation of a large series of cases to characterise this aggressive subtype of invasive lobular carcinoma. This subtype, which includes the pleomorphic and high-grade solid variants accounts for 14% of lobular carcinomas. It showed associations with unfavourable prognostic features, poor patient outcomes, and poor response to therapy when compared to classic lobular carcinoma and invasive ductal carcinoma of no special type.

**Abstract:**

Background: Invasive lobular carcinoma (ILC), the most common special type of breast cancer (BC), has unique clinical behaviour and is different from invasive ductal carcinoma of no special type (IDC-NST). However, ILC further comprises a diverse group of tumours with distinct features. This study aims to examine the clinicopathological and prognostic features of different variants of ILC, with a particular focus on characterising aggressive subtypes. Methods: A large (n = 7140) well-characterised and histologically reviewed BC cohort with treatment and long-term follow-up data was investigated. The cohort was classified based on the WHO classification of tumours into main histological subtypes, including ILC and IDC-NST. ILCs were further classified into variants. Clinicopathological parameters and patient outcomes in terms of BC-specific survival (BCSS) and disease-free survival (DFS) were evaluated. Results: ILC constituted 11% of the cohort. The most common non-classic ILC variants were pleomorphic (pILC) and solid (sILC), constituting 19% of ILC. Compared to classic and related variants (alveolar, trabecular, papillary, and tubulolobular; cILC), pILC and sILC variants were associated with aggressive tumour characteristics. The histologic grade of ILC was an important prognostic variable. The survival patterns identified an aggressive ILC subtype encompassing pILC and high-grade sILC. These tumours, which comprised 14% of the cases, were associated with clinicopathological characteristics of poor prognosis and had high BC-specific death and recurrence rates compared not only to cILC (*p* < 0.001) but also to IDC-NST (*p* = 0.02) patients. Contrasting this, cILC patients had significantly longer BCSS and DFS than IDC-NST patients in the first 10 to 15 years of follow-up. Adjuvant chemotherapy did not improve the outcome of patients with aggressive ILC subtypes. Conclusions: pILC and high-grade sILC variants comprise an aggressive ILC subtype associated with poor prognostic characteristics and a poor response to chemotherapy. These results warrant confirmation in randomised clinical trials.

## 1. Introduction

Invasive lobular carcinoma (ILC) is the second most common breast cancer (BC) histological type after invasive ductal carcinoma of no special type (IDC-NST) [1]. In recent years, the incidence of ILC has increased from 5% to 10–15% due to improved diagnostic and surveillance approaches [2].

ILC has distinct morphological features, molecular signatures, and clinical behaviour that are different from those of IDC-NST and other special types of BC [3]. Loss of E-cadherin function, diffuse growth pattern, and low proliferative activity, in addition to oestrogen receptor (ER) expression (>90%) and human epidermal growth factor 2 (HER2) negativity, are characteristic features of ILC [4,5,6]. ILC also shows unique metastatic patterns with involvement of sites less commonly involved by IDC-NST [7], including the gastrointestinal tract, with the stomach being the most commonly affected site [8], genitourinary tract, retroperitoneum, and peritoneum [9]. Early metastatic ILC also tends to be infiltrative rather than mass-forming [10]. Although classical ILC is characterised by cells with small nuclei and scanty cytoplasm, a dyscohesive growth pattern, arrangement in single strands with minimal stromal reaction, and infiltration around breast ductal units (targetoid lesions) [6,11], cytological and architectural variations exist. ILC cells may exhibit a high degree of pleomorphism, warranting the diagnosis of a pleomorphic ILC variant (pILC) or displaying diffuse solid infiltration in the solid variant (sILC). ILC cells may also be arranged in tubules, clusters, or trabeculae in tubulolobular, alveolar, and trabecular variants, respectively [4,12,13].

As IDC-NST is the most common type of BC, accounting for more than 60% of cases, it has steered the majority of BC research, and differences between various histological types have been less investigated [3]. For a long time, patients with ILC were treated using the same protocols used for IDC-NST. However, recent studies have shown that ILC patients respond less to chemotherapeutic agents [14,15,16]. Therefore, there is now greater awareness of the different invasive BC subtypes, including the unique characteristics of ILC, and a move towards personalised treatment approaches.

Although most of the previous studies have reported the clinical behaviour of ILC based on the most common variant, classical ILC (cILC), and on comparison between the cILC and IDC-NST [2,17,18,19,20,21], there is increasing evidence that some variants of ILC have distinct and aggressive clinical behaviour. pILC is the variant of ILC associated with negative prognostic factors, mainly ER negativity and HER2 positivity [22,23]. However, the consideration of pILC as a separate entity and its impact on patient management remains controversial, and most patients with pILC are managed in the same way as cILC [24]. Similarly, some authors have reported that sILC is an aggressive, highly proliferative variant of ILC compared with cILC [12]; however, studies addressing the characteristics of this variant remain lacking.

Previous studies have demonstrated the poor response of ILC patients to neoadjuvant chemotherapy [3,12,25,26]. However, the response of ILC variants to neoadjuvant therapies remains unclear [27]. Moreover, it is unclear if the difference in the clinical behaviour and response to therapy of ILC variants is related to the degree of proliferation (histological grade), receptor status, or the histological subtypes of ILC [28].

Therefore, this study aims to characterise ILC variants for management purposes utilising a large well-characterised cohort of BC with long-term follow-up coupled with a detailed histological review.

## 2. Materials and Methods

### 2.1. Study Cohort

A well-characterised consecutive cohort (n = 7140) of patients presented to Nottingham City Hospital from 1998 to 2018 with early-stage (TNM cT1-2, cN0-3, M0) operable BC was investigated. Patients with locally advanced or metastatic BC or those who did not undergo surgery for any reason or received neoadjuvant therapy were not included in this study. Loco-regional radiological staging was performed using ultrasound, but MRI was used in cases diagnosed as ILC on the core biopsy according to local protocol. Clinicopathological data, including patient age, tumour histological type, tumour size, tumour grade and its components, lymph node (LN) status, lymphovascular invasion (LVI), and Nottingham Prognostic Index (NPI), were retrieved from the data repository. Biomarker expression data, including ER, progesterone receptor (PR), HER2, and Ki67 index, were collected from patient reports. Hormone receptors and HER2 were scored according to updated UK guidelines and American Society of Clinical Oncology/College of American Pathologists (ASCO/CAP) guidelines [29,30,31]. Ki67 > 14% was considered a high index [32]. Oncotype Dx recurrence scores (RS) were also available in a subset of tumours (n = 431). RS of <11, 11–25, and >25 were defined as low, intermediate, and high, respectively [33]. Follow-up data, including BC-specific survival (BCSS), defined as the time from the initial surgery to death related to BC, and disease-free survival (DFS), defined as the time from the initial surgery to the development of any recurrence event of disease, in addition to the site of recurrences, were collected.

Adjuvant systemic therapies were given following a multidisciplinary team decision according to ER status, NPI, menopausal status, and associated comorbidities. ER-positive BC patients with good prognostic NPI received endocrine therapy (ET). Chemotherapy regimens were given to ER-negative patients. ER + BC patients with moderate and poor prognostic NPI (>4.4) received ET with combined chemotherapy if patients were fit to tolerate chemotherapy. In later years of the study, HER2 status, Oncotype Dx RS, and TNM stage were also considered in the decision of adjuvant therapy (A subset of patients with NPI 3.4–4.4 were tested using the Oncotype Dx assay for adjuvant chemotherapy, and most HER2 positive patients receive adjuvant Herceptin in addition to chemotherapy). ET was administered as the only adjuvant therapy in 47% of the cohort; 14% of patients received only chemotherapy; and 14% received both ET and chemotherapy. Adjuvant radiotherapy was applied according to local protocols.

### 2.2. Invasive Lobular Carcinoma Histological Assessment

All cases diagnosed as ILC in routine practice, where histological slides were available (72% of cases), were reviewed by a certified pathologist (SM). In Nottingham, the diagnosis of lobular type and variants is based on morphology, while E-cadherin use is limited to occasional cases that show overlapping features between ILC and ductal carcinoma [34]. None of the cases was designated as pILC in this study unless it was histologically reviewed, and the classification was approved by all the observers. Variants were classified according to the 5th edition of the WHO Classification of Tumours [35] into classic, pleomorphic, solid, tubulolobular, alveolar, trabecular, histiocytoid, and signet ring. Mixed variants encompassing more than one variant were reclassified and assigned the potentially aggressive variant. In cases of discordance with the original reports, discordant cases were reviewed by other experienced pathologists (ER and NA) until a consensus was reached.

**Figure 1:**
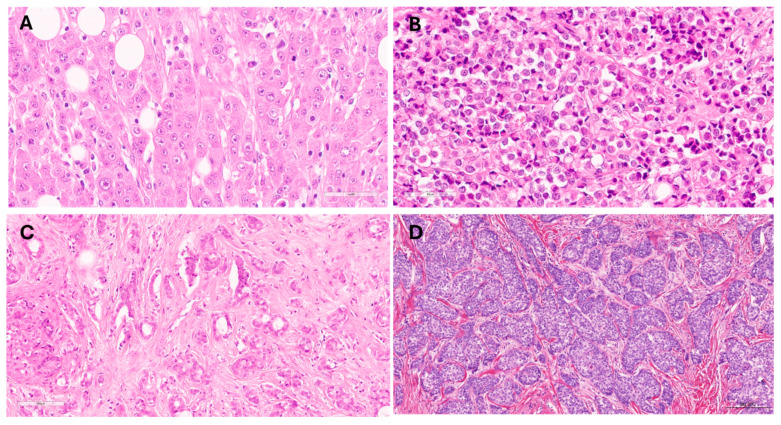
Examples of non-classic variants of invasive lobular carcinoma (ILC). (**A**) Pleomorphic ILC (scale bar: 60 µm); (**B**) Solid ILC (scale bar: 60 µm); (**C**) Tubulo-lobular carcinoma (scale bar: 100 µm); and (**D**) Alveolar ILC (scale bar: 200 µm).

cILC exhibited small to medium-sized dyscohesive cells arranged in single files or targetoid patterns with low to intermediate-grade nuclei. ILC cells with marked cellular pleomorphism with or without apocrine morphology were classified as pILC. sILC displayed a solid growth pattern of small lobular cells arranged in large, solid sheets with scarce intervening stroma. sILC often mimics IDC-NST but lacks polarisation of the cells, which are E-cadherin negative. The few cases with solid growth patterns but marked cellular pleomorphism were classified as pILC. If the solid growth pattern shows papillary architecture, ILC is classified as papillary ILC. Arrangement in broad trabeculae or clusters of at least 20 cells was diagnosed as trabecular and alveolar variants, respectively. When cells exhibit abundant granular foamy cytoplasm, histiocytoid differentiation is recognised, while signet ring carcinoma features intracytoplasmic accumulation resembling signet ring cells [3,4,6,11,35,36,37] (Figure 1). In routine practice, E-cadherin immunohistochemistry was used in cases with overlapping features, and in such a case where the tumour was E-cadherin negative, the classification of ILC was confirmed. In cases with classic morphology, the classification of ILC was confirmed without using E-cadherin, as 10% of ILC with classic morphology show E-cadherin membrane expression [34].

### 2.3. Statistical Analysis

Statistical Package SPSS v28 for Windows (Chicago, IL, USA) was used. The chi-square test was used to compare categorical groups. Inter-observer agreement was determined using the intra-class correlation coefficient (ICC). Kaplan–Meier curves and log-rank tests were used for survival analysis. The mean patient follow-up was 127 months. A *p*-value of less than 0.05 (two-tailed) was considered significant in all the statistical tests.

## 3. Results

### 3.1. Patient and Tumour Characteristics

Eleven percent of the cohort was classified as ILC, 63% as IDC-NST, and 26% as other special or mixed histological types. The mean patient age was 57 years (ranging from 32 to 87), 59 years in ILC, and 56 years in IDC-NST. Eighteen percent of the cohort was classified as grade 1, while 43% and 39% were grade 2 and 3 tumours, respectively. High-grade (grade 3) tumours comprised 56% of IDC-NST and 8% of ILC. ER was positive in 80% of the cohort, 96% in ILC, and 71% in IDC-NST.

The mean tumour size of the cohort was 19 mm. However, ILC had a mean size of 23 mm, while IDC-NST had a mean size of 19 mm. LN metastasis was detected in 34% of the whole cohort, 33% of ILC, and 35% of IDC-NST patients. However, when the analysis was limited to grade-matched cases, the frequency of LN positivity was 33% in ILC compared to 31% in IDC-NST. Of the LN-positive patients, the mean number of positive LN was 3.9 in ILC and 3.0 in IDC-NST. In this early-stage cohort, 54% of patients were treated with breast-conserving therapy, 47% of ILC patients, and 53% of patients with IDC-NST.

### 3.2. Invasive Lobular Carcinoma Variants

In this study, a few cases were classified as tubulolobular, trabecular, alveolar, and papillary variants, and these were classified as grade 1 and 2 tumours, had small lobular carcinoma cells, and were combined with the classic variant as related variants. The classic and related variants (cILC) comprised 81% of ILC, while 19% of ILC exhibited pleomorphic, solid, histiocytoid, and signet ring morphologies (Figure 1). pILC and sILC constituted most of the non-classic variants (67% and 29%, respectively). The histiocytoid and signet ring variants were insufficiently represented for meaningful statistical analyses and were excluded from subsequent outcome analyses.

### 3.3. Correlation between ILC Variants and Clinicopathological Characteristics

pILC was significantly associated with poor tumour characteristics, including a higher histological grade, a larger tumour size, LN metastasis, a higher probability of LVI, a poorer NPI prognostic group, ER and PR negativity, HER2 overexpression, a higher Ki67 index, and a younger patient age compared to cILC (Table 1). Compared to IDC-NST, pILC revealed a significant association with larger tumour size, LN metastasis, and poorer NPI; however, it displayed a lower histological grade, mitotic count, and Ki67 index than IDC-NST.

sILC showed a significantly higher histological grade, mitotic count, and Ki67 index than cILC. However, sILC had a lower histological grade and mitotic count than IDC-NST (Table 1). Comparing the two variants, pleomorphic and solid, pILC was significantly associated with a higher grade, larger tumour size, LN metastasis, and poorer NPI than sILC. However, a higher Ki67 index was shown in the solid variant than in the pleomorphic variant.

In contrast to the aggressive features seen in pILC and sILC, cILC was associated with favourable characteristics compared to IDC-NST. cILC was more frequently associated with older age, lower grade and grade components, and good NPI. Moreover, cILC was more frequently of low- and intermediate-risk Oncotype Dx RS than IDC-NST (Table 1).

### 3.4. Outcome Analyses

**Figure 2:**
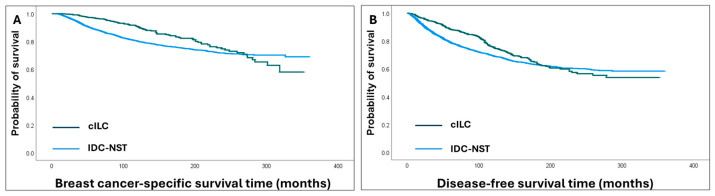
Kaplan–Meier survival analysis shows initial favourable outcomes of the classic invasive lobular carcinoma (cILC) subtype compared to invasive ductal carcinoma of no special type (IDC-NST) that decreased over time, with the survival curves tending to cross at 15 years: (**A**) breast cancer-specific survival; (**B**) disease-free survival.

cILC patients displayed BC-specific death in 14% and recurrence in 26% of patients, compared to 19% and 29%, respectively, of IDC-NST patients. Patients with the cILC subtype had longer BCSS (HR = 0.7, 95% CI = 0.5–0.8, *p* < 0.001) and DFS (HR = 0.8, 95% CI = 0.7–0.9, *p* = 0.008) compared to IDC-NST patients. However, cILC continued to develop events over time, and the significance decreased at 10–15 years (Figure 2).

The clinicopathological characteristics and survival patterns of ILC variants were further examined to identify the aggressive, high-risk ILC variants. As both pILC and sILC showed aggressive features compared to cILC, we aimed to determine whether to include the whole variants or stratify them based on a combination of variant and grade as histological grade played a significant prognostic role (A significantly shorter BCSS was observed in patients with grade 3 ILC subtype compared to those with grade 2 pILC; HR = 2.2, 95% CI = 1.1–4.6, *p* = 0.04).

Grade 2 pILC was associated with significantly younger patient age, larger tumour size, and LVI than cILC (Supplementary Table S1). With a mean patient follow-up of 127 months, the BC-specific death of pILC was 28%, while 43% of pILC patients experienced recurrence. Within grade 2 pILC, BC-specific death and recurrence were seen in 23% and 40% of the cases, respectively. In grade 3 pILC, 34% and 46% of patients experienced BC-specific death and recurrence, respectively.

Grade 2 sILC did not show significant differences from cILC, apart from the characteristic higher proliferation manifested in the mitotic score and Ki-67 index. sILC patients experienced BC-specific death in 19% of cases and recurrence in 29% of cases. However, grade stratification revealed a more aggressive survival pattern associated with grade 3 sILC, where 22% and 33% of patients died and developed recurrence, respectively, compared to 18% and 27%, respectively, of grade 2 sILC. When compared with cILC patients, no outcome differences were identified for grade 2 sILC, whereas significant BCSS and DFS differences were identified between cILC and pILC grades (*p* < 0.001) (Supplementary Figure S1).

Therefore, pILC, where both grades displayed aggressive behaviour, and grade 3 sILC patients, that were associated with higher risk, were combined in one group ‘aggressive ILC subtype’, which comprised 14% of ILC and was further analysed.

The aggressive ILC subtype was associated with large tumour size, positive LN metastasis (46%), high grade (grade 3 in 50%), and a high Ki-67 index. Over 80% had NPI scored as moderate and poor prognostic groups. All characteristics are summarised in Table 2.

**Figure 3:**
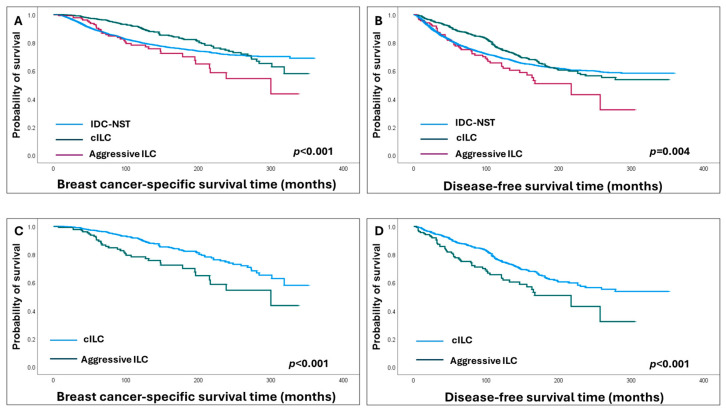
Kaplan–Meier survival analysis shows (**A**,**B**) survival outcomes of invasive lobular carcinoma (ILC) subtypes compared to invasive ductal carcinoma of no special type (IDC-NST). (**C**,**D**) Poor survival outcomes are associated with aggressive ILC compared to cILC.

Statistically significant outcome differences were observed between ILC subtypes and IDC-NST (*p* = 0.001 and *p* = 0.004, for BCSS and DFS, respectively) (Figure 3).

Compared to cILC, patients with the aggressive ILC subtype suffered significantly shorter BCSS and DFS (HR = 2.2, 95% CI = 1.4–3.4, *p* < 0.001, and HR = 1.8, 95% CI = 1.3–2.5, *p* < 0.001). Moreover, worse DFS was associated with an aggressive ILC subtype compared to IDC-NST patients (HR = 1.4, 95% CI = 1.1–1.9, *p* = 0.02) (Figure 3).

Multivariate Cox regression analyses involving the ILC subtypes (classic versus aggressive) and adjusted for known prognostic parameters: NPI, ER status, and HER2 status revealed an independent prognostic significance for shorter BCSS (HR = 1.7, 95% CI = 1.1–2.7, *p* = 0.01) and shorter DFS (HR = 1.5, 95% CI = 1.1–2.1, *p* = 0.02) associated with aggressive ILC subtype. The same results were associated with the aggressive ILC subtype compared to IDC-NST (HR = 1.6, 95% CI = 1.1–2.3, *p* = 0.006 for BCSS, HR = 1.5, 95% CI = 1.2–2.0, *p* = 0.003 for DFS) in a multivariate model. However, cILC could not retain a significant difference from IDC-NST when tested in multivariate analyses (Supplementary Table S2).

**Figure 4:**
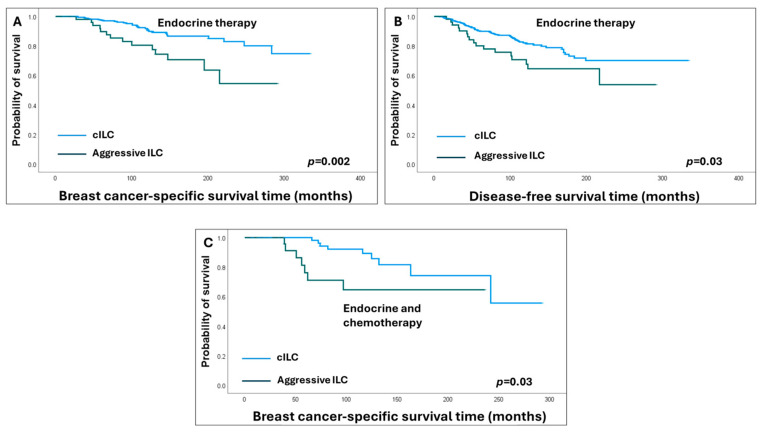
Kaplan–Meier survival analysis shows shorter survival outcomes of aggressive invasive lobular carcinoma (ILC) subtypes compared to classic ILC (cILC) in endocrine-treated only (**A**,**B**) and combined endocrine and chemotherapy-treated (**C**) patients.

In the context of adjuvant therapies, patients with aggressive ILC subtypes who received only adjuvant ET exhibited worse survival outcomes compared to those with cILC (HR = 2.8, 95% CI = 1.5–5.2, *p* = 0.002 for BCSS and HR = 1.8, 95% CI = 1.1–3.1, *p* = 0.03 for DFS). Similar results were obtained when survival analyses were restricted to patients who received adjuvant ET and chemotherapy, where patients with aggressive ILC subtypes remained to show shorter BCSS (HR = 2.9, 95% CI = 1.1–8, *p* = 0.03) compared to cILC patients (Figure 4).

**Figure 5:**
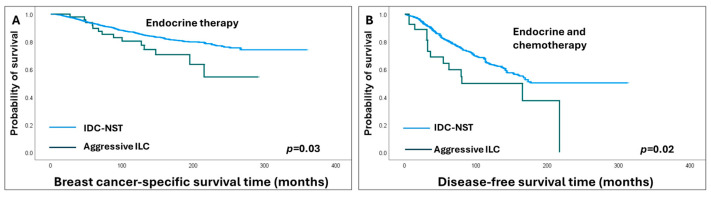
Kaplan–Meier survival curves show poor survival outcomes of aggressive invasive lobular carcinoma (ILC) compared to invasive ductal carcinoma of no special type (IDC-NST) in endocrine-treated only (**A**) and combined endocrine and chemotherapy-treated patients (**B**).

Patients with aggressive ILC subtypes also demonstrated shorter BCSS (HR = 1.8, 95% CI = 1.1–3.0, *p* = 0.03) compared to IDC-NST patients when both were treated with adjuvant ET alone and shorter DFS (HR = 1.9, 95% CI = 1.1–3.2, *p* = 0.02) when treated with adjuvant ET and chemotherapy (Figure 5).

**Figure 6:**
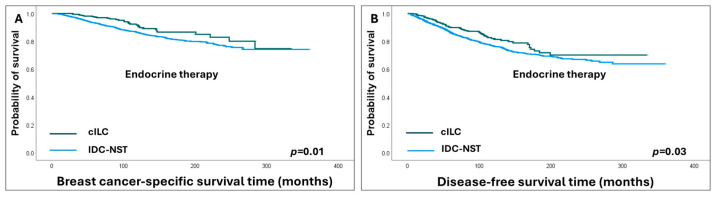
Kaplan–Meier survival curves show favourable survival outcomes of classic invasive lobular carcinoma (cILC) compared to invasive ductal carcinoma of no special type (IDC-NST) in endocrine-treated patients: (**A**) breast cancer-specific survival; (**B**) disease-free survival.

Compared to IDC-NST, cILC patients showed a favourable response to adjuvant ET (HR = 0.6, 95% CI = 0.4–0.9, *p* = 0.01 and HR = 0.7, 95% CI = 0.6–0.9, *p* = 0.03 for BCSS and DFS, respectively) (Figure 6), but when adjuvant chemotherapy was given, IDC-NST patients’ outcomes improved and the survival differences between cILC and IDC-NST patients became insignificant.

## 4. Discussion

The most common special histological subtype, ILC, has shown a rise in incidence in recent years because of the significant advancements in imaging modalities [6]. The unique characteristic morphology and behaviour of ILC have captivated the clinical interest in this special subtype [11]. However, the clinical outcomes of ILC vary widely in the literature and are conflicting, mandating further investigation in large, well-characterised cohorts [12].

Investigating a large, well-characterised, and histologically reviewed cohort of early-stage (cT1-2) operable BC patients who were treated uniformly in Nottingham with long-term follow-up (>20 years), we demonstrated favourable survival outcomes of cILC compared to IDC-NST. Nevertheless, a tendency toward BC-specific death and recurrence was noticed at 15 years. These findings are in line with Chamalidou et al. [17], where an excess mortality rate ratio was calculated and more favourable outcomes of ILC than IDC-NST were observed at 5 years while significantly decreasing at 10–15 years after diagnosis and then similar after 20 years [17].

Worse outcomes associated with ILC than IDC-NST were reported [18,20,38], while others revealed similar outcomes [1,2,19,21,39,40]. This variation in outcome findings is likely to reflect the variability in the ILC cohorts with variable representations of ILC histological variants [2,3,17]. Therefore, we aimed to refine the prognosis of ILC variants. We identified various morphological variants, where pILC and sILC comprised 19% of our cohort. Few studies addressed the prognostic relevance of ILC variants, which revealed comparable proportions [12,27].

Thorough analyses of the survival patterns of pILC and sILC could identify a subgroup of ILC with aggressive behaviour. By stratifying ILC variants based on histological grade, none of the cILC were classified as grade 3 tumours. Both grades of pILC seemed to behave worse than cILC, while only grade 3 sILC was more aggressive than cILC. We combined these two tumour types into one group that was termed the “aggressive ILC subtype”. The aggressive subtype accounts for 14% of ILC cases, requiring further clinical research and potentially contributing to over 1000 cases annually in the UK based on recent BC statistics [41].

This subtype showed poorer outcomes not only when compared to the classic ILC and related variants (cILC) but also to IDC-NST. Poor tumour characteristics were observed in the aggressive ILC subtypes, including larger tumour size, younger patient age, LN metastasis, higher proliferative activity, and more frequent ER negativity and HER2 positivity. These findings confirm the aggressive behaviour stated by previous studies [13,22,23] and are in line with previous studies on pILC [12,22,42]. pILC was previously reported to have worse overall survival than IDC-NST [13], similar to the shorter survival associated with the aggressive ILC subtype observed in our study. However, there is a debate about considering pILC as a standalone and independent prognostic variable and whether it is a variant or grade that should be considered [23,24,43]. It is worth noting that pILC has a distinct genetic profile compared to cILC. The former is more likely to have a *TP53* mutation (11–42% of pILC, compared to less than 10% of cILC), as well as complex DNA copy number alterations, FER kinase expression, altered DNA methylation patterns, and mutations in both *IRS2* and *IGFR* [44,45].

The clinical behaviour of sILC is less defined, and only a limited number of cases were studied, which showed an association between sILC and shorter survival compared to other ILC variants [12]. This study found that sILC is associated with higher grades, pleomorphism, mitosis, and ki-67 proliferation index than cILC, in addition to an association with worse outcomes. Therefore, sILC was further investigated, and this showed that the worse outcome compared to cILC was limited to grade 3 tumours. Other ILC variants, including histiocytoid and signet-ring morphologies, were reported as aggressive variants [46,47,48,49]. However, the data about these variants are scarce and were excluded from our analyses due to their limited number.

As a predominantly ER + BC subtype, ILC patients are offered ET, while the response to chemotherapy is said to be less sensitive [6]. The same survival patterns for ILC and its variants were present, as expected when endocrine-treated patients were selected, while the high-risk chemotherapy-treated ILC patients were not responsive compared to IDC-NST. Chemotherapy did not seem beneficial for improved survival outcomes in several reports [2,14,16,50].

However, in our study, aggressive ILC subtypes were associated with a worse chemotherapy response than classic variants and IDC-NST. The poor response of aggressive ILC to both ET and chemotherapies revealed in this study confirms the need for further large studies and trials investigating additional therapies tailored to these specific subtypes.

Response to neoadjuvant chemotherapy was previously reported to be higher in IDC-NST than ILC, which confirms the decreased sensitivity of ILC to chemotherapeutic agents. pCR rates ranged in previous reports between 0–11% in ILC patients while reaching up to 70% in IDC-NST patients [25,26,27,51] as the pCR rate varies widely according to the molecular subtype [52]. There is a debate on whether the low pCR rates in ILC versus IDC-NST are due to differences in ER expression, where ILC, which was ER-negative and/or HER2+, had a pCR rate of 25% [25,53]. A previous study demonstrated that the pCR rate was 4.2% in ER-positive, grade 1 or 2 ILC, 7.0% in ILC with either ER-negative or grade 3, and 17.8% in ER-negative grade 3 ILC [54]. When the variant of ILC was considered, Mamtani and colleagues [27] demonstrated a higher pCR of pILC than cILC patients (16% of pILC compared to only 1% of cILC).

Evaluation of Oncotype Dx RS in ILC histology has been tried previously, where the consistent association of ILC to low RS was revealed [50,55,56], supporting our results. Weiser et al. [57] found a role for RS in predicting ILC prognosis; however, our cohort’s survival data for Oncotype Dx are insufficient to confirm these results.

The study has limitations. First, the cohort is retrospective, and further validation in a randomised clinical trial is warranted. Second, the rarity of some ILC variants, mainly the signet ring cell variant and the histiocytoid variant, did not allow for studying their clinical behaviour and whether these tumours should be combined with cILC or the aggressive subtype.

## 5. Conclusions

In conclusion, ILC encompasses a mixture of variants with different clinical behaviours. pILC and high-grade sILC variants comprise an aggressive ILC subtype. Aggressive ILC variants are associated with poor prognostic characteristics, are at higher risk of recurrence and BC-related death than cILC patients, and show a poor response to chemotherapy. The poor response of the aggressive ILC group to chemotherapy requires further investigation for innovative and tailored therapies.

## Figures and Tables

**Table 1:** Correlation of invasive lobular carcinoma compared to invasive ductal carcinoma of no special type to clinicopathological parameters.

Characteristics	Pleomorphic Invasive Lobular Carcinoma (pILC) N (%)	Solid Invasive Lobular Carcinoma (sILC) N (%)	Classic Invasive Lobular Carcinoma (cILC) N (%)	Invasive Ductal Carcinoma, No Special Type (IDC-NST) N (%)	X^2^ (*p*-Value) ^a^ pILC vs. cILC	X^2^ (*p*-Value) ^b^ pILC vs. IDC-NST	X^2^ (*p*-Value) ^c^ sILC vs. cILC	X^2^ (*p*-Value) ^d^ sILC vs. IDC-NST	X^2^ (*p*-Value) ^e^ pILC vs. sILC	X^2^ (*p*-Value) ^f^ cILC vs. IDC-NST
**Age at diagnosis (years)** <50 ≥50	27 (22) 69 (78)	6 (14) 36 (86)	95 (16) 503 (84)	1366 (30) 3109 (70)	8.6 **(0.003)**	0.26 (0.6)	0.07 (0.7)	5.2 **(0.02)**	3 (0.07)	55.1 **(<0.001)**
**Tumour size (cm)** <2 ≥2	29 (30) 67 (70)	20 (48) 22 (52)	344 (57) 254 (43)	2672 (60) 1797 (40)	24.8 **(<0.001)**	34.0 **(<0.001)**	1.6 (0.2)	2.6 (0.1)	3.9 **(0.04)**	1.1 (0.2)
**Tumour grade** 1 2 3	0 52 (54) 44 (46)	0 33 (79) 9 (21)	55 (9) 543 (91) 0	320 (7) 1659 (37) 2496 (56)	295.9 **(<0.001)**	15.9 **(<0.001)**	38.8 **(<0.001)**	7.9 **(0.005)**	7.4 **(0.007)**	687.4 **(<0.001)**
**Mitotic count** 1 2 3	52 (54) 28 (29) 16 (17)	26 (62) 7 (17) 9 (21)	563 (94) 35 (6) 0	1612 (36) 957 (21) 1906 (43)	156.7 **(<0.001)**	26.2 **(<0.001)**	95.5 **(<0.001)**	12.5 **(0.002)**	2.3 (0.2)	734.8 **(<0.001)**
**Nuclear pleomorphism** 1 2 3	0 0 96 (100)	0 42 (100) 0	30 (5) 568 (95) 0	14 (1) 1066 (23) 3395 (76)	522.9 **(<0.001)**	29.8 **(<0.001)**	2 (0.1)	122.1 **(<0.001)**	138 **(<0.001)**	1412.1 **(<0.001)**
**Tubule formation** 1 2 3	0 0 96 (100)	0 0 42 (100)	6 (1) 40 (7) 552 (92)	88 (2) 1120 (25) 3267 (73)	7.2 **(0.007)**	32.6 **(<0.001)**	3.2 (0.07)	14.3 **(<0.001)**	-	106.3 **(<0.001)**
**Nottingham Prognostic Index** Good Prognostic Group Moderate Prognostic Group Poor Prognostic Group	19 (20) 52 (54) 25 (26)	15 (36) 25 (59) 2 (5)	317 (53) 239 (40) 41 (7)	1288 (29) 2453 (55) 719 (16)	54.2 **(<0.001)**	8.4 **(0.01)**	2.4 (0.1)	4.2 (0.1)	9.9 **(0.007)**	149.5 **(<0.001)**
Axillary nodal status * Negative Positive	51 (53) 45 (47)	31 (74) 11 (26)	411 (69) 186 (31)	2886 (65) 1580 (35)	9.1 **(0.002)**	5.4 **(0.02)**	0.5 (0.5)	1.5 (0.2)	5.2 **(0.02)**	4.1 **(0.04)**
**Lymph node stage** 1 (Negative) 2 (1–3 positive) 3 (>3 positive)	51 (53) 29 (30) 16 (17)	31 (74) 9 (21) 2 (5)	411 (69) 131 (22) 55 (9)	2886 (65) 1206 (27) 374 (8)	10.0 **(0.007)**	9.8 **(0.007)**	0.8 (0.3)	1.7 (0.1)	6 **(0.04)**	6.9 **(0.03)**
**Lymphovascular invasion** Negative Positive	67 (70) 29 (30)	34 (81) 8 (19)	536 (90) 62 (10)	3241 (72) 1234 (28)	28.6 **(<0.001)**	0.33 (0.5)	2.6 (0.1)	1.6 (0.2)	1.9 (0.1)	82.1 **(<0.001)**
Distant metastasis site ** Common Uncommon	34 (97) 1 (3)	8 (89) 1 (11)	96 (91) 10 (9)	947 (98) 18 (2)	1.9 (0.1)	0.16 (0.6)	0.02 (0.8)	1.9 (0.1)	0.9 (0.3)	14.1 **(<0.001)**
**Oestrogen receptor** Negative Positive	13 (13) 83 (87)	0 42 (100)	12 (2) 576 (98)	1297 (30) 3103 (70)	31 **(<0.001)**	11.6 **(<0.001)**	1.7 (0.1)	17.5 **(<0.001)**	6.3 **(0.01)**	201.7 **(<0.001)**
**Progesterone receptor** Negative Positive	34 (37) 59 (63)	8 (20) 31 (80)	135 (25) 413 (75)	1834 (44) 2318 (56)	5.8 **(0.01)**	2.1 (0.1)	0.3 (0.5)	8.8 **(0.003)**	3 (0.07)	75.9 **(<0.001)**
**Human epidermal growth factor receptor 2** Negative Positive	81 (91) 8 (9)	38 (97) 1 (3)	542 (99) 8 (1)	3359 (81) 766 (19)	17.8 **(<0.001)**	5.3 **(0.02)**	0.3 (0.6)	6.6 (0.1)	2.0 (0.1)	102.9 **(<0.001)**
**Ki67 index** Low (≤14%) High (>14%)	28 (54) 24 (46)	7 (29) 17 (71)	220 (79) 60 (21)	744 (35) 1394 (65)	14.2 **(<0.001)**	8.1 **(0.005)**	28.5 **(<0.001)**	0.3 (0.5)	4.0 **(0.04)**	197.9 **(<0.001)**
**Oncotype Dx recurrence score** Low Intermediate High	2 (22) 6 (67) 1 (1)	4 (36) 6 (55) 1 (9)	5 (11) 37 (82) 3 (7)	44 (16) 160 (56) 80 (28)	0.2 (0.6)	1.2 (0.2)	2.0 (0.1)	4.0 (0.1)	0.5 (0.7)	12.0 **(0.003)**
**Breast surgery** Breast-conserving Mastectomy	35 (36) 61 (64)	23 (55) 19 (45)	279 (47) 319 (53)	2354 (53) 2121 (47)	3.5 (0.06)	9.8 **(0.002)**	1.0 (0.3)	0.08 (0.7)	4.0 **(0.04)**	7.5 **(0.006)**
**Endocrine therapy** No Yes	24 (25) 72 (75)	4 (10) 38 (90)	161 (27) 433 (73)	1727 (39) 2711 (61)	0.19 (0.6)	7.7 **(0.006)**	6.3 **(0.01)**	15.2 **(<0.001)**	4.3 **(0.03)**	31.7 **(<0.001)**
**Chemotherapy** No Yes	61 (64) 35 (36)	38 (90) 4 (10)	504 (84) 94 (16)	2849 (64) 1625 (36)	23.5 **(<0.001)**	0.001 (0.9)	1.2 (0.2)	13 **(<0.001)**	10.5 **(0.001)**	99.9 **(<0.001)**

* Routine IHC for LN examination is not routinely performed, which may lead to underestimated nodal metastasis. ** Common metastatic sites include lymph nodes, lung, liver, bone, and brain. Uncommon sites include gastrointestinal, ovarian, urinary tract, endometrial, and orbital metastases. ^a^ Comparison between pILC and cILC variants; ^b^ Comparison between pILC and IDC-NST; ^c^ Comparison between sILC and cILC; ^d^ Comparison between sILC and IDC-NST; ^e^ Comparison between pILC and sILC; ^f^ Comparison between cILC and IDC-NST. Significant *p*-values are in bold.

**Table 2:** Clinicopathological characteristics of the aggressive invasive lobular carcinoma subtype (n = 105).

Characteristics	Frequency (%)
Age at diagnosis (years)	
<50	29 (28)
≥50	76 (72)
Tumour size (cm) <2 ≥2	33 (31) 72 (69)
Tumour grade	
1	0
2	52 (50)
3	53 (50)
Mitotic count	
1	52 (49)
2	28 (27)
3	25 (24)
Nuclear pleomorphism	
1	0
2	9 (9)
3	96 (91)
Tubule formation	
1	0
2	0
3	100 (100)
Nottingham Prognostic Index	
Good Prognostic Group	19 (18)
Moderate Prognostic Group	60 (57)
Poor Prognostic Group	26 (25)
Lymph node stage	
1 (Negative)	57 (54)
2 (1–3 positive)	31 (30)
3 (>3 positive)	17 (16)
Lymphovascular invasion	
Negative	74 (70)
Positive	31 (30)
Oestrogen receptor	
Negative	13 (12)
Positive	92 (88)
Progesterone receptor	
Negative	34 (34)
Positive	67 (66)
HER2	
Negative	89 (92)
Positive	8 (8)
Ki67 index	
Low (≤14%)	28 (48)
High (>14%)	30 (52)
Oncotype Dx recurrence score	
Low	2 (20)
Intermediate	6 (60)
High	2 (20)
Breast surgery	
Breast-conserving	39 (37)
Mastectomy	66 (63)
Endocrine therapy	
No	24 (23)
Yes	81 (77)
Chemotherapy	
No	69 (66)
Yes	36 (34)

## Data Availability

The data presented in the current study are available upon reasonable request.

## Supplementary Materials

The following supporting information can be downloaded at: https://www.mdpi.com/article/10.3390/cancers16101893/s1, Supplementary Figure S1: Kaplan Meier survival curves show shorter breast cancer-specific survival (A) and disease-free survival (B) associated with pleomorphic invasive lobular carcinoma (pILC) compared to classic ILC (cILC); Supplementary Table S1: Clinicopathological characteristics of grade 2 pleomorphic invasive lobular carcinoma (pILC) and grade 2 solid ILC (sILC) in comparison to classic ILC (cILC); Supplementary Table S2: Multivariate Cox Regression analysis shows prognostic variables for breast cancer-specific survival and disease-free survival.
