# Supplementary material for: Deciphering the Clinical Behaviour of Invasive Lobular Carcinoma of the Breast Defines an Aggressive Subtype
Source: Cancers (Basel). 2024 May 16;16(10):1893. doi: 10.3390/cancers16101893 (PMC11120092; doi:10.3390/cancers16101893)
Supplement: Supplementary file 1 [file cancers-16-01893-s001.zip › cancers-3023439-supplementary.pdf]

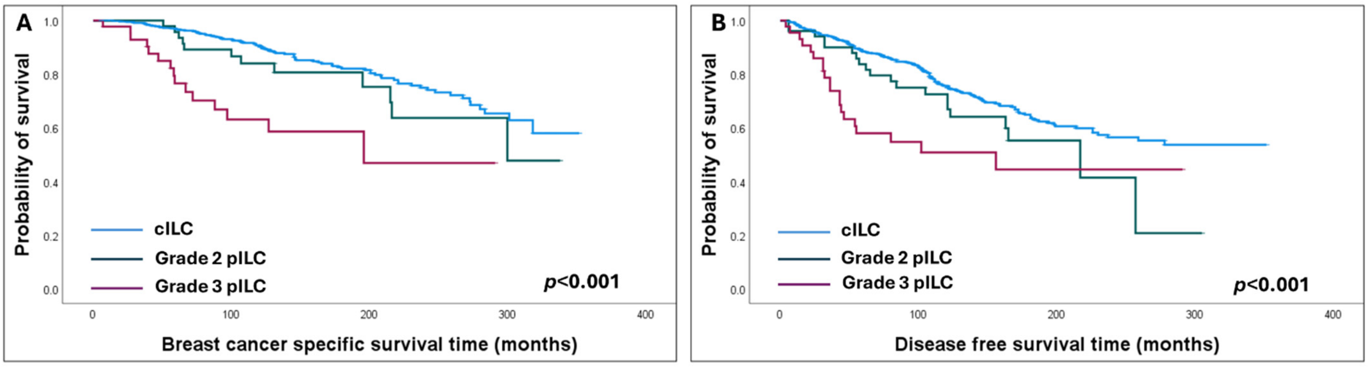

Supplementary Figure S1. Kaplan Meier survival curves show shorter breast cancer-specific survival (A) and disease-free survival (B) associated with pleomorphic invasive lobular carcinoma (pILC) compared to classic ILC (cILC).

Supplementary Table S1. Clinicopathological characteristics of grade 2 pleomorphic invasive lobular carcinoma (pILC) and grade 2 solid ILC (sILC) in comparison to classic ILC (cILC).

**Characteristics**	**Grade 2 pILC N (%)**	**Grade 2 sILC N (%)**	**cILC N (%)**	**X^2 (*p*-value) [a]**	**X^2 (*p*-value) [b]**
**Age at diagnosis (years)**					
< 50	18 (35)	4 (12)	95 (16)	11.7	0.3
≥ 50	34 (65)	29 (88)	503 (84)	**(<0.001)**	(0.5)
**Tumour size (cm)**					
< 2	20 (38)	16 (49)	344 (57)	7.1	1
≥ 2	32 (62)	17 (51)	254 (43)	**(0.008)**	(0.3)
**Mitotic count**					
1	52	26 (79)	563 (94)	3.1	8.4
2	0	7 (21)	35 (6)	(0.07)	**(0.01)**
3	0	0	0		
**Nuclear pleomorphism**					
1	0	0	30 (5)	415.3	1.6
2	0	33 (100)	568 (95)	**(<0.001)**	(0.2)
3	52	0	0		
**Tubule formation**					
1	0	0	6 (1)	8	5.1
2	0	0	40 (7)	**(0.01)**	(0.07)
3	52 (100)	33 (100)	552 (92)		
**Nottingham Prognostic Index**					
Good Prognostic Group	19 (36)	15 (45)	317 (53)	5.5 (0.06)	2.2 (0.3)
Moderate Prognostic Group	29 (56)	17 (52)	239 (40)		
Poor Prognostic Group	4 (8)	1 (3)	41 (7)		
**Lymph node stage**					
1 (Negative)	31 (59)	25 (76)	411 (69)	2.1	2.0
2 (1-3 positive)	16 (36)	7 (21)	131 (22)	(0.3)	(3.7)
3 (>3 positive)	5 (10)	1 (3)	55 (9)		
**Lymphovascular invasion**					
Negative	40 (77)	27 (82)	536 (90)	7.7	1.7
Positive	12 (23)	6 (18)	62 (10)	**(0.006)**	(0.1)
**Distant metastasis site***					
Common	17 (100)	6 (86)	96 (91)	3.1	0.2
Uncommon	0	1 (4)	10 (9)	(0.07)	(0.6)
**Oestrogen receptor**					
Negative	5 (10)	0	12 (2)	6.8	1.3
Positive	47 (90)	33 (100)	576 (98)	**(0.009)**	(0.2)

**Progesterone receptor**					
Negative	14 (27)	8 (26)	135 (25)	0.2	0.02
Positive	37 (73)	23 (74)	413 (75)	(0.6)	(0.8)
**HER2**					
Negative	47 (96)	30 (97)	542 (99)	1.4	0.5
Positive	2 (4)	1 (3)	8 (1)	(0.2)	(0.4)
**Ki67 index**					
Low (≤14%)	22 (79)	7 (39)	220 (79)	0.1	12.2
High (>14%)	6 (21)	11 (61)	60 (21)	(1)	**(<0.001)**
**Oncotype Dx recurrence score**					
Low	1 (20)	4 (40)	5 (11)	0.9	4.9
Intermediate	4 (80)	6 (60)	37 (82)	(0.6)	(0.08)
High	0	0	3 (7)		
**Breast surgery**					
Breast-conserving	24 (46)	19 (58)	279 (47)	0.005	1.5
Mastectomy	28 (54)	14 (42)	319 (53)	(0.9)	(0.4)
**Endocrine therapy**					
No	17 (33)	4 (12)	161 (27)	0.7	3.6
Yes	35 (67)	29 (78)	433 (73)	(0.3)	(0.05)
**Chemotherapy**					
No	36 (69)	30 (91)	504 (84)	7.7	1.1
Yes	16 (31)	3 (9)	94 (16)	**(0.005)**	(0.3)

[a] Comparison between grade 2 pILC and cILC.

[b] Comparison between grade 2 sILC and cILC.

Supplementary Table S2. Multivariate Cox Regression analysis shows prognostic variables for breast cancer-specific survival and disease-free survival.

**Feature**		**Breast cancer-specific survival**			**Disease free survival**		
		**Hazard ratio**	**95% Confidence interval**	***P*-value**	**Hazard ratio**	**95% Confidence interval**	***P*-value**
**A**	**ILC subtype** (Aggressive vs classic)	1.7	1.1-2.7	**0.01**	1.5	1.1-2.1	**0.02**
	**Nottingham Prognostic Index** (Poor vs good and intermediate)	3.6	2.2-5.8	**<0.001**	3.3	3.2-4.8	**<0.001**
	**Oestrogen receptor status** (ER- vs ER+)	2.2	1.1-4.5	**0.03**	1.7	0.9-3.2	0.1
	**HER2 status** (HER2+ vs HER2-)	1.1	0.4-3.2	0.8	1	0.4-2.3	0.9
**B**	**Histological subtype** (Aggressive ILC vs IDC-NST)	1.6	1.1-2.8	**0.006**	1.5	1.2-2.0	**0.003**
	**Nottingham Prognostic Index** (Poor vs good and intermediate)	3.2	2.8-3.7	**<0.001**	2.6	2.3-2.9	**<0.001**
	**Oestrogen receptor status** (ER- vs ER+)	1.5	1.3-1.7	**<0.001**	1.4	1.2-1.6	**<0.001**
	**HER2 status** (HER2+ vs HER2-)	1.2	1.1.-1.4	**0.02**	1.2	1.1-1.4	**0.01**
**C**	**Histological subtype** (cILC vs IDC-NST)	0.6	0.7-1.2	0.5	1	0.8-1.2	0.9
	**Nottingham Prognostic Index** (Poor vs good and intermediate)	3.3	2.8-3.8	**<0.001**	2.6	2.3-2.9	**<0.001**
	**Oestrogen receptor status** (ER- vs ER+)	1.4	1.3-1.7	**<0.001**	1.4	1.2-1.6	**<0.001**
	**HER2 status** (HER2+ vs HER2-)	1.2	1.1-1.4	**0.03**	1.2	1.1-1.4	**0.01**

cILC; classic invasive lobular carcinoma, IDC-NST; invasive ductal carcinoma of no special type.

Significant *p*-values are in bold.
